# Supplementary material for: Conserved lipid metabolic reprogramming confers hypoxic and aging resilience
Source: EMBO Rep. 2025 Dec 11;27(3):704–28. doi: 10.1038/s44319-025-00664-6 (PMC12894929; doi:10.1038/s44319-025-00664-6)
Supplement: Supplementary file 2 — Table EV2 [file 44319_2025_664_MOESM2_ESM.docx]

| Strains | SOURCE | IDENTIFIER |
| --- | --- | --- |
| *C. elegans*: N2 (wild type) | CGC | N/A |
| *C. elegans*:  *fasn-1(g14)* | CGC | GG14 |
| *C. elegans*:  *fat-6(tm331); fat-5(tm420)* | CGC | BX110 |
| *C. elegans*:  *fat-6p::fat-6::GFP* | This paper | DMS2136 |
| *C. elegans*:  *fasn-1::gfp* | CGC | AG400 |
| *C. elegans*:  *cysl-2p::Venus* | Ma lab | DMS640 |
| *C. elegans*: *dhs-3p::dhs-3::GFP* | CGC | LIU86 |
| *C. elegans*: *rpl-28p::mCherry::pdr-1* | Ma lab | DMS1110 |
| *C. elegans*: *rab-3p::ND18ApoE4* | CGC | JPS844 |
| *C. elegans*: *hsp-16.2p::GFP* | Ma lab | DMS63 |
| *C. elegans*: *rab-3p::ND18ApoE4; hsp-16.2p::GFP* | Ma lab | DMS2298 |
| *C. elegans*: *ser-2prom-3p::myr-GFP* | Shen lab | TV15911 |
| *C. elegans*: *rab-3p::ND18ApoE4; ser-2prom-3p::myr-GFP* | Ma lab | DMS2320 |
| *C. elegans*: *mai-2p::mai-2::GFP* | González lab | RN80 |
| *C. elegans*: *unc-54p::Q40::YFP* | CGC | AM141 |
| *C. elegans*: *rab-3p::ND18ApoE4; unc-54p::Q40::YFP* | Ma lab | DMS2390 |
| *C. elegans*: *unc-129p::ctns-1::mCherry + nlp-21p::Venus + ttx-3p::RFP]* | CGC | KG2430 |
| *C. elegans*: *rab-3p::ND18ApoE4; unc-129p::ctns-1::mCherry + nlp-21p::Venus + ttx-3p::RFP]* | Ma lab | DMS2470 |

Genotypes of strains used:
