# Supplementary figures and images for: Conserved lipid metabolic reprogramming confers hypoxic and aging resilience
Source: EMBO Rep. 2025 Dec 11;27(3):704–28. doi: 10.1038/s44319-025-00664-6 (PMC12894929; doi:10.1038/s44319-025-00664-6)

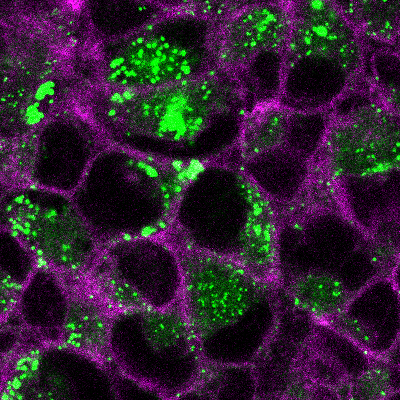

Supplement: Supplementary file 6 — Source data Fig. 1 [file 44319_2025_664_MOESM6_ESM.zip › Figure 1/1E/AGS NSCs.tif]

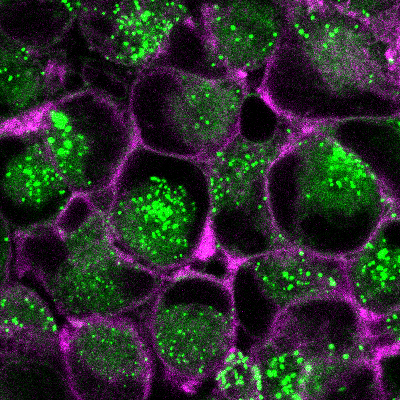

Supplement: Supplementary file 6 — Source data Fig. 1 [file 44319_2025_664_MOESM6_ESM.zip › Figure 1/1E/Mouse NSCs.tif]

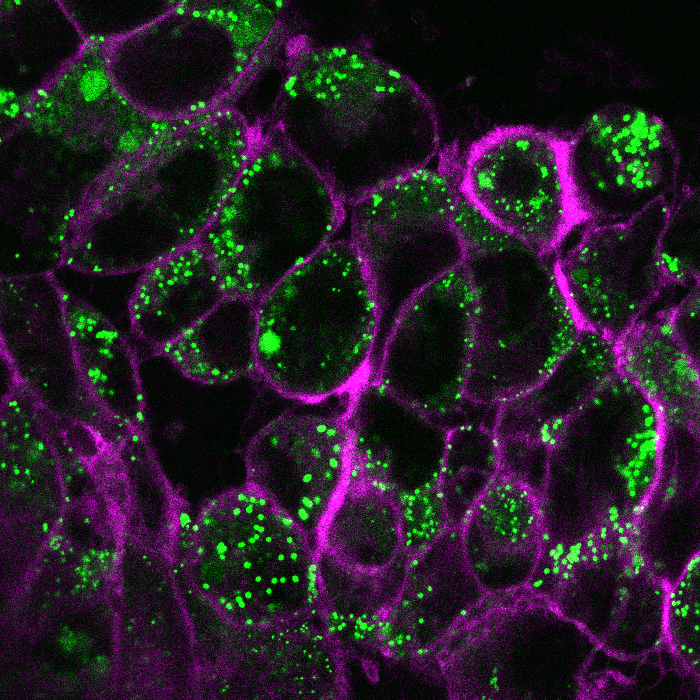

Supplement: Supplementary file 8 — Source data Fig. 3 [file 44319_2025_664_MOESM8_ESM.zip › Figure 3/3A/HEK293T Merged.tif]

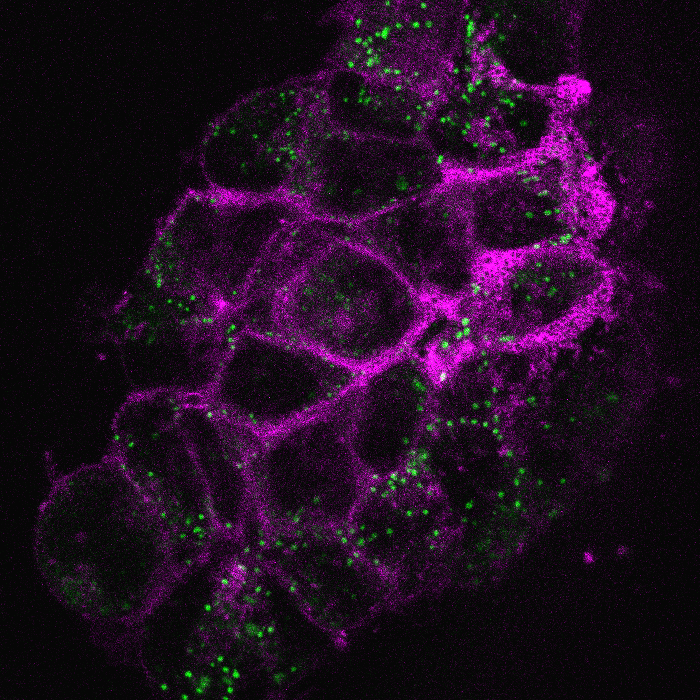

Supplement: Supplementary file 8 — Source data Fig. 3 [file 44319_2025_664_MOESM8_ESM.zip › Figure 3/3A/Non-degradable HIF-1 Merged.tif]

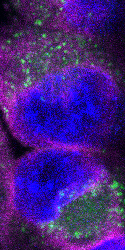

Supplement: Supplementary file 8 — Source data Fig. 3 [file 44319_2025_664_MOESM8_ESM.zip › Figure 3/3B/Cerulenin Mag.tif]

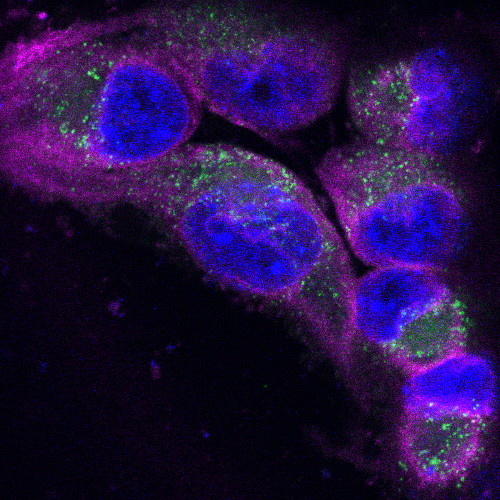

Supplement: Supplementary file 8 — Source data Fig. 3 [file 44319_2025_664_MOESM8_ESM.zip › Figure 3/3B/Cerulenin Merged.tif]

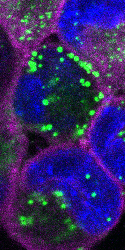

Supplement: Supplementary file 8 — Source data Fig. 3 [file 44319_2025_664_MOESM8_ESM.zip › Figure 3/3B/DMSO Mag.tif]

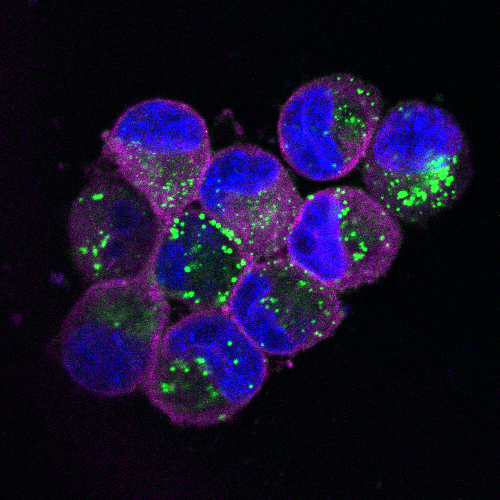

Supplement: Supplementary file 8 — Source data Fig. 3 [file 44319_2025_664_MOESM8_ESM.zip › Figure 3/3B/DMSO Merged.tif]

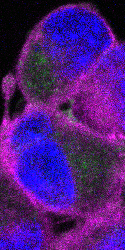

Supplement: Supplementary file 8 — Source data Fig. 3 [file 44319_2025_664_MOESM8_ESM.zip › Figure 3/3B/TOFA Mag.tif]

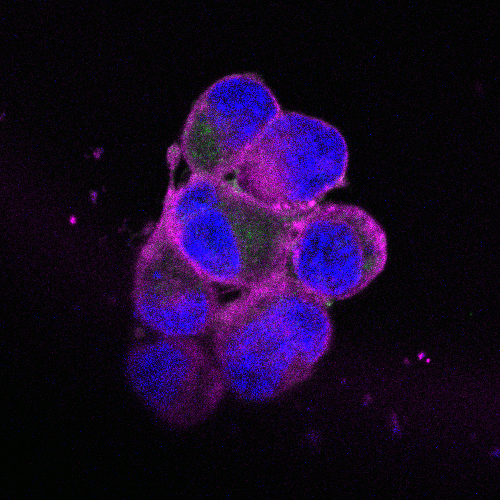

Supplement: Supplementary file 8 — Source data Fig. 3 [file 44319_2025_664_MOESM8_ESM.zip › Figure 3/3B/TOFA Merged.tif]

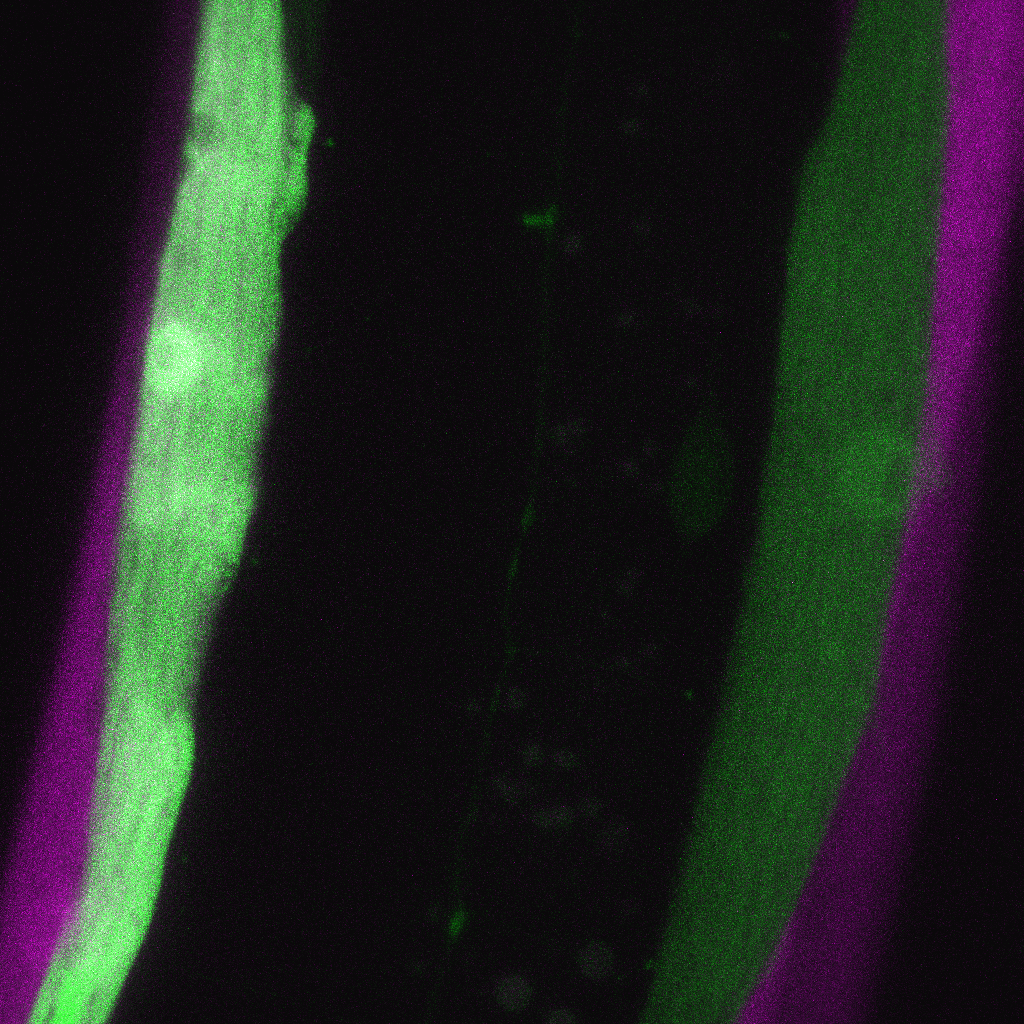

Supplement: Supplementary file 10 — Source data Fig. 5 [file 44319_2025_664_MOESM10_ESM.zip › Figure 5/5A/APOE4-merged DMSO Hig mag.tif]

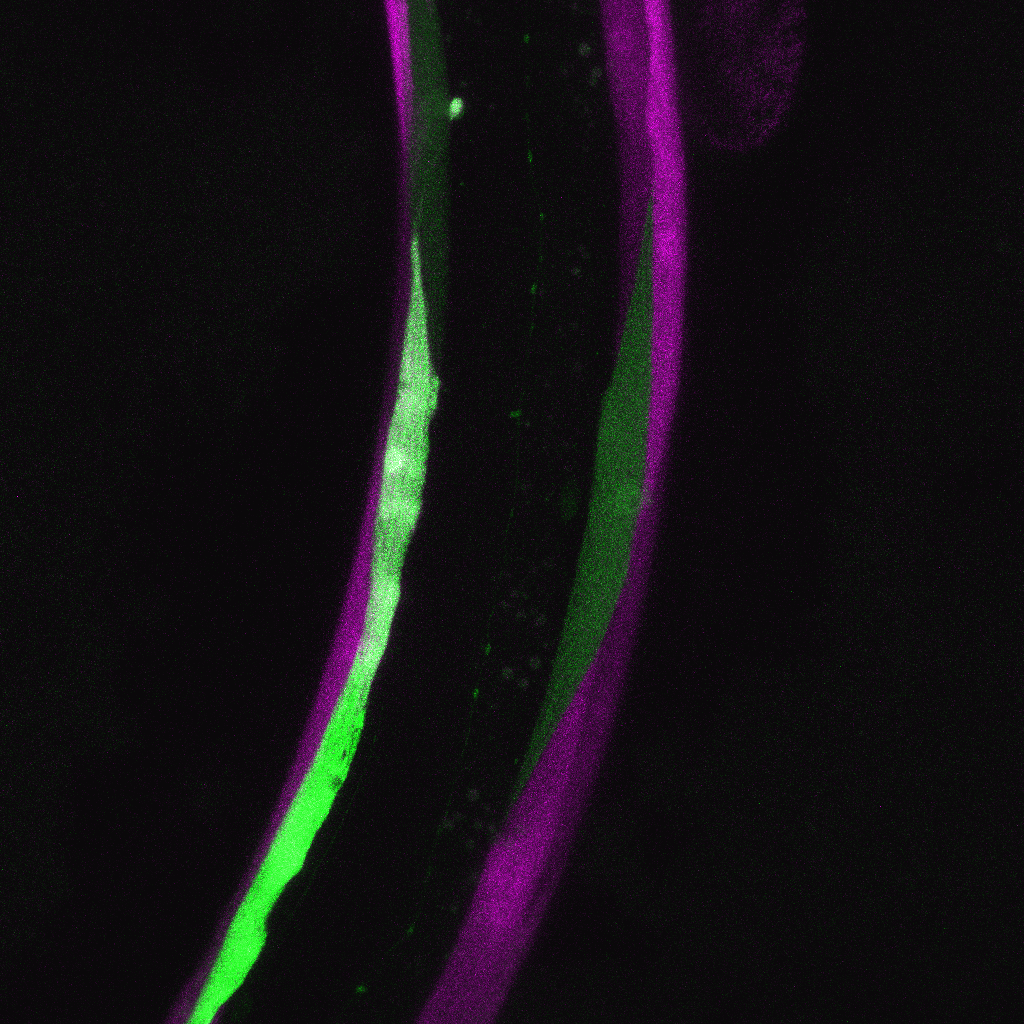

Supplement: Supplementary file 10 — Source data Fig. 5 [file 44319_2025_664_MOESM10_ESM.zip › Figure 5/5A/APOE4-merged DMSO Low mag.tif]

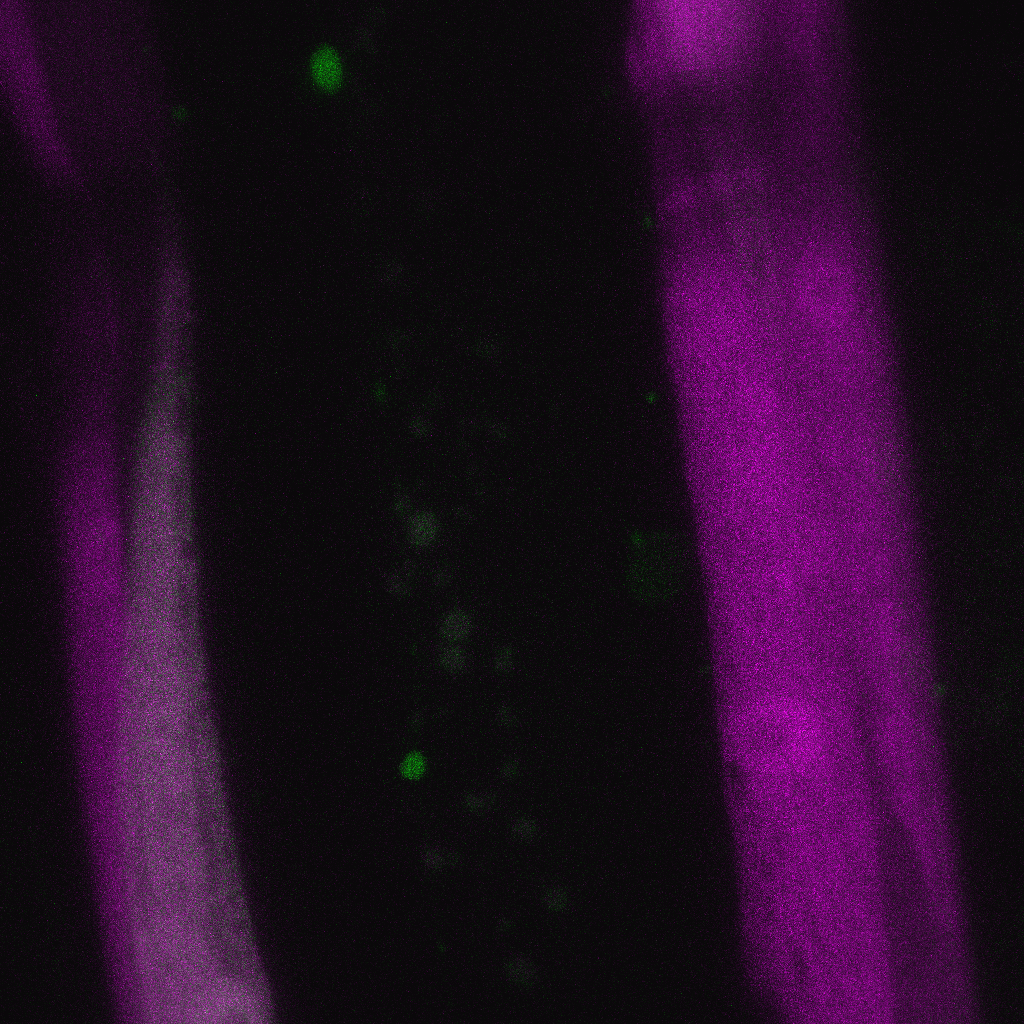

Supplement: Supplementary file 10 — Source data Fig. 5 [file 44319_2025_664_MOESM10_ESM.zip › Figure 5/5A/APOE4-merged TOFA Hig mag.tif]

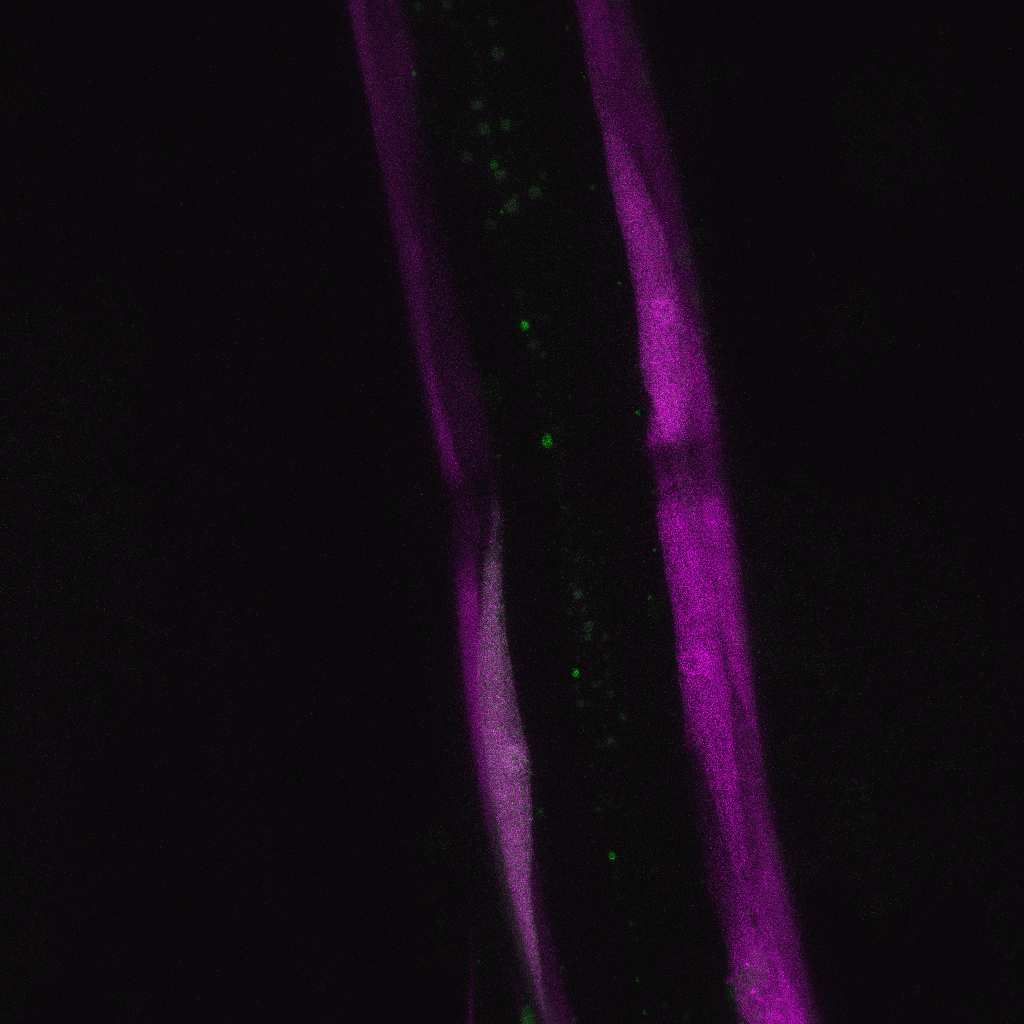

Supplement: Supplementary file 10 — Source data Fig. 5 [file 44319_2025_664_MOESM10_ESM.zip › Figure 5/5A/APOE4-merged TOFA Low mag.tif]

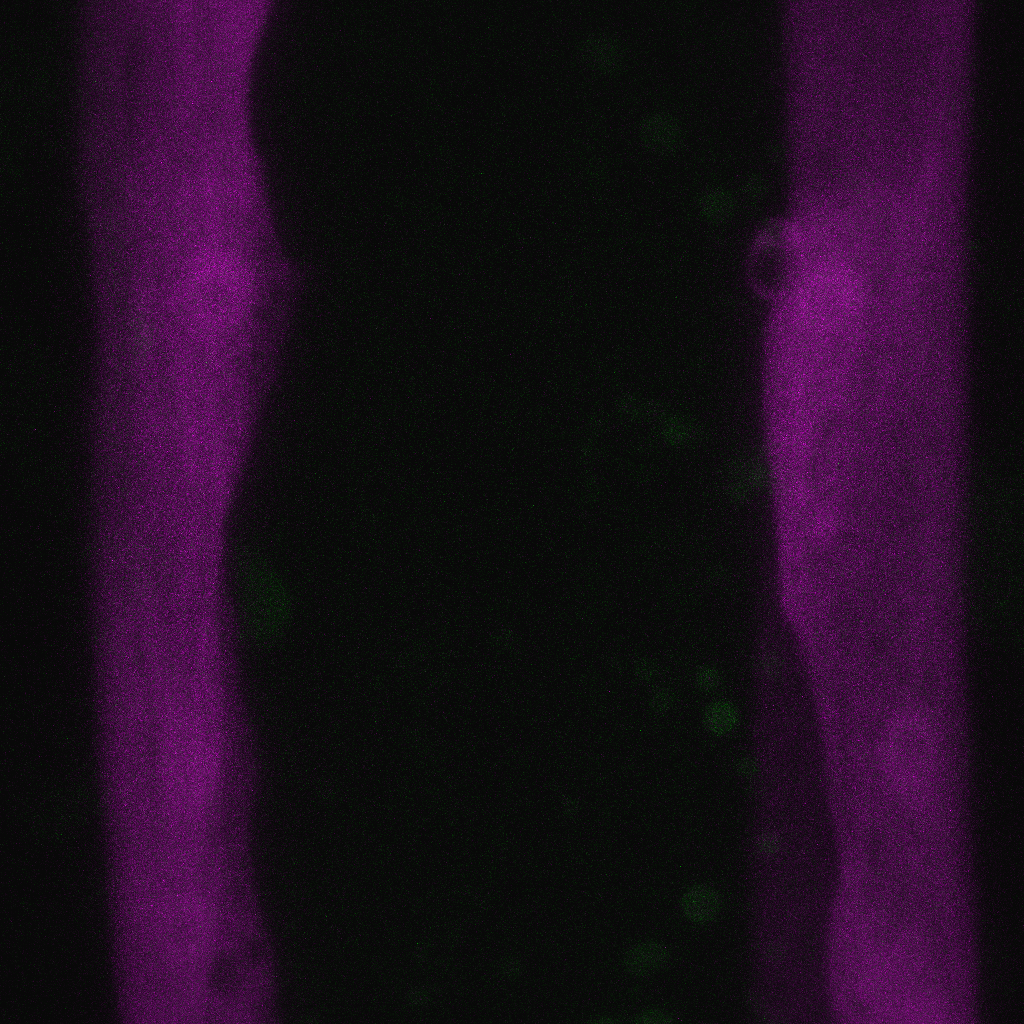

Supplement: Supplementary file 10 — Source data Fig. 5 [file 44319_2025_664_MOESM10_ESM.zip › Figure 5/5A/DMSO Merged Hig mag.tif]

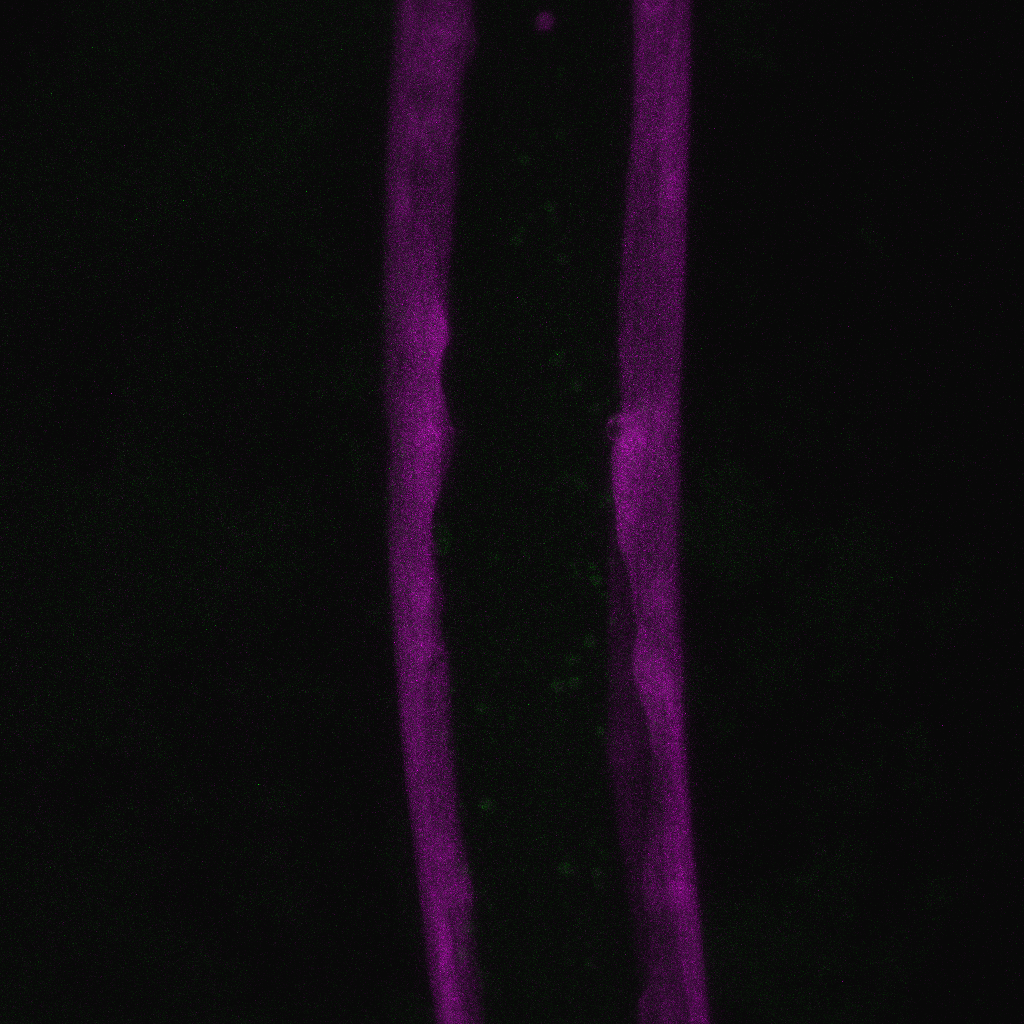

Supplement: Supplementary file 10 — Source data Fig. 5 [file 44319_2025_664_MOESM10_ESM.zip › Figure 5/5A/DMSO Merged Low mag.tif]

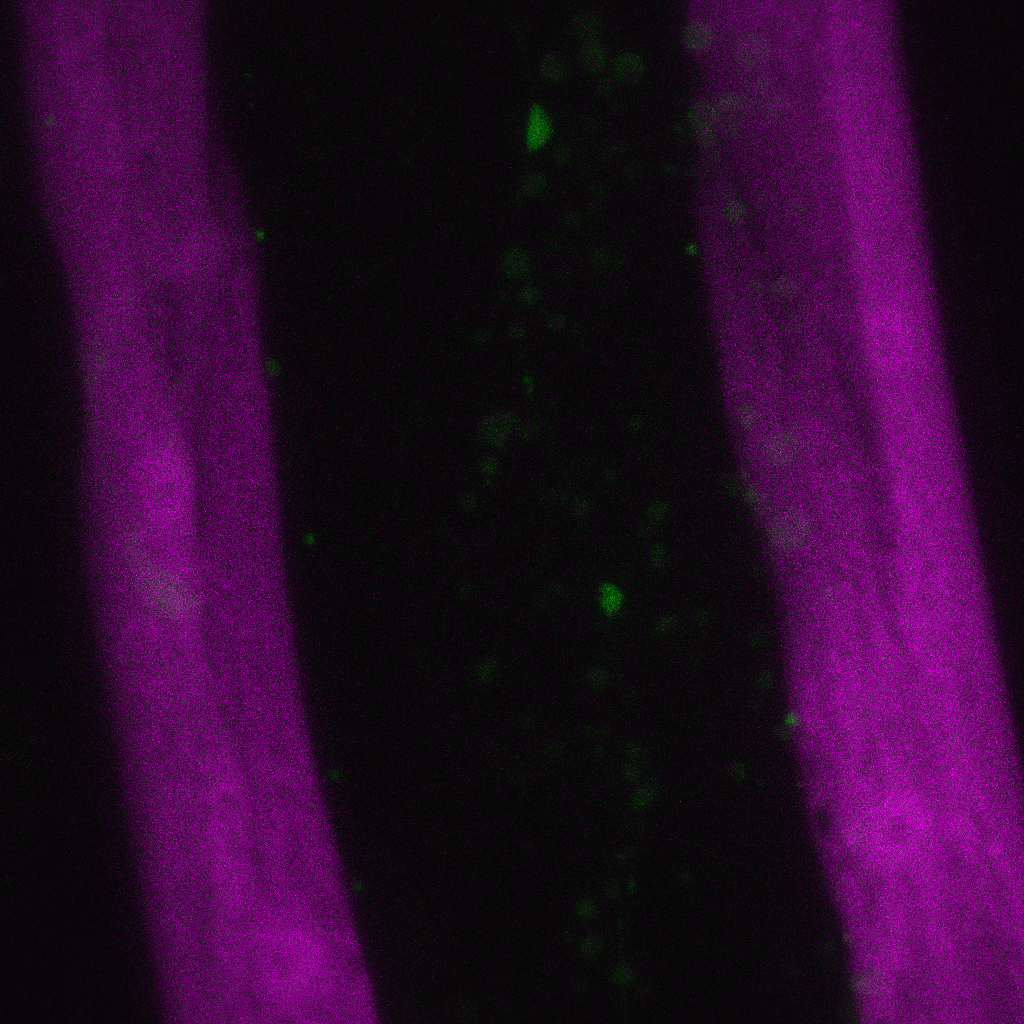

Supplement: Supplementary file 10 — Source data Fig. 5 [file 44319_2025_664_MOESM10_ESM.zip › Figure 5/5A/TOFA Merged Hig mag.tif]

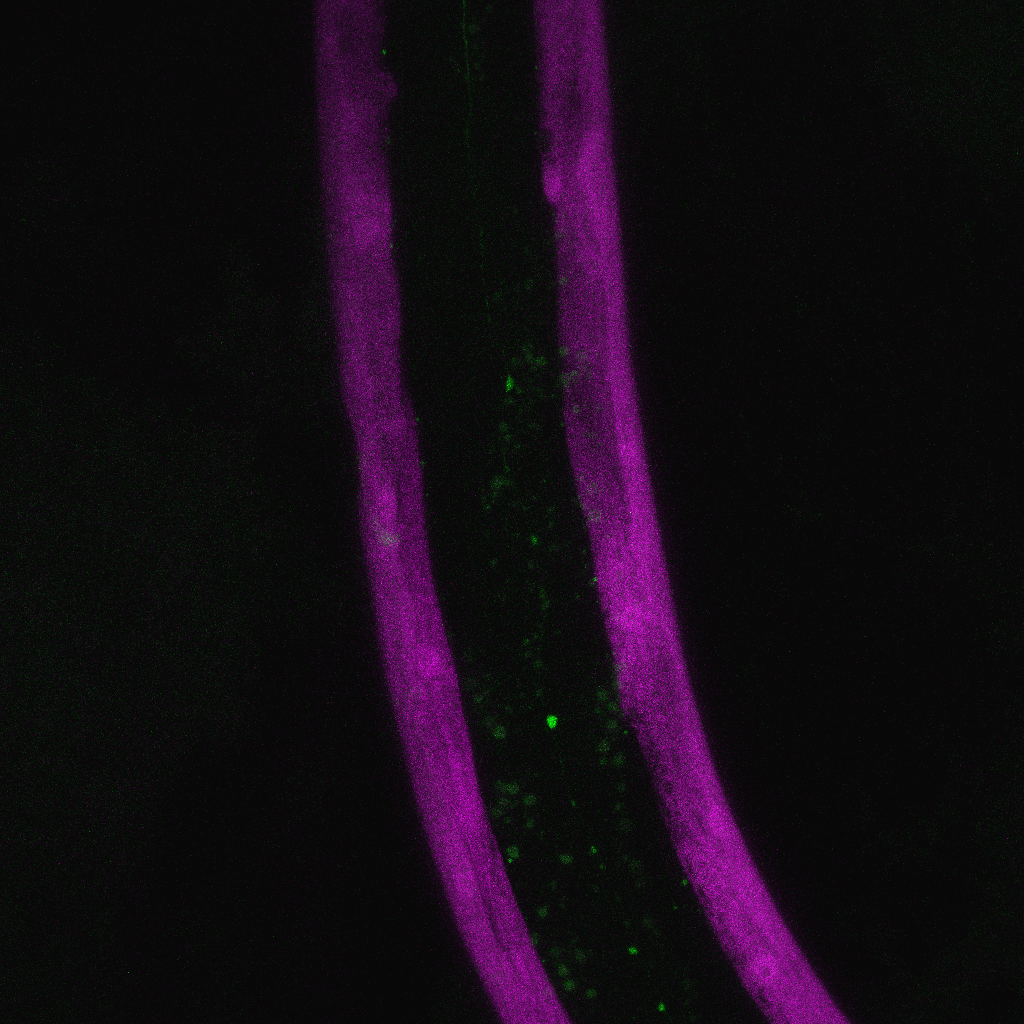

Supplement: Supplementary file 10 — Source data Fig. 5 [file 44319_2025_664_MOESM10_ESM.zip › Figure 5/5A/TOFA Merged Low mag.tif]
