# Supplementary material for: Conserved lipid metabolic reprogramming confers hypoxic and aging resilience
Source: EMBO Rep. 2025 Dec 11;27(3):704–28. doi: 10.1038/s44319-025-00664-6 (PMC12894929; doi:10.1038/s44319-025-00664-6)
Supplement: Supplementary file 12 — Expanded View Figures [file 44319_2025_664_MOESM12_ESM.pdf]

Expanded View Figures

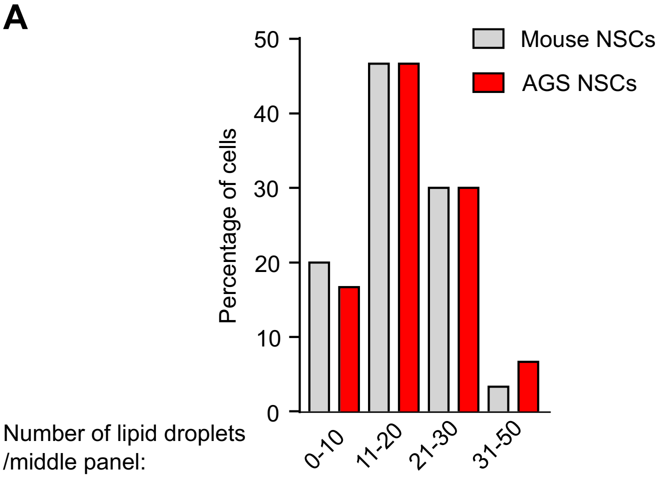

Figure EV1. Characterization of lipid droplets in AGS NSCs and mouse NSCs under normal conditions.

(**A**) Quantification of the percentage of cells with varying numbers of lipid droplets per mid-plane image in mouse and AGS NSCs ($n > 25$ cells per condition).

 © The Author(s)

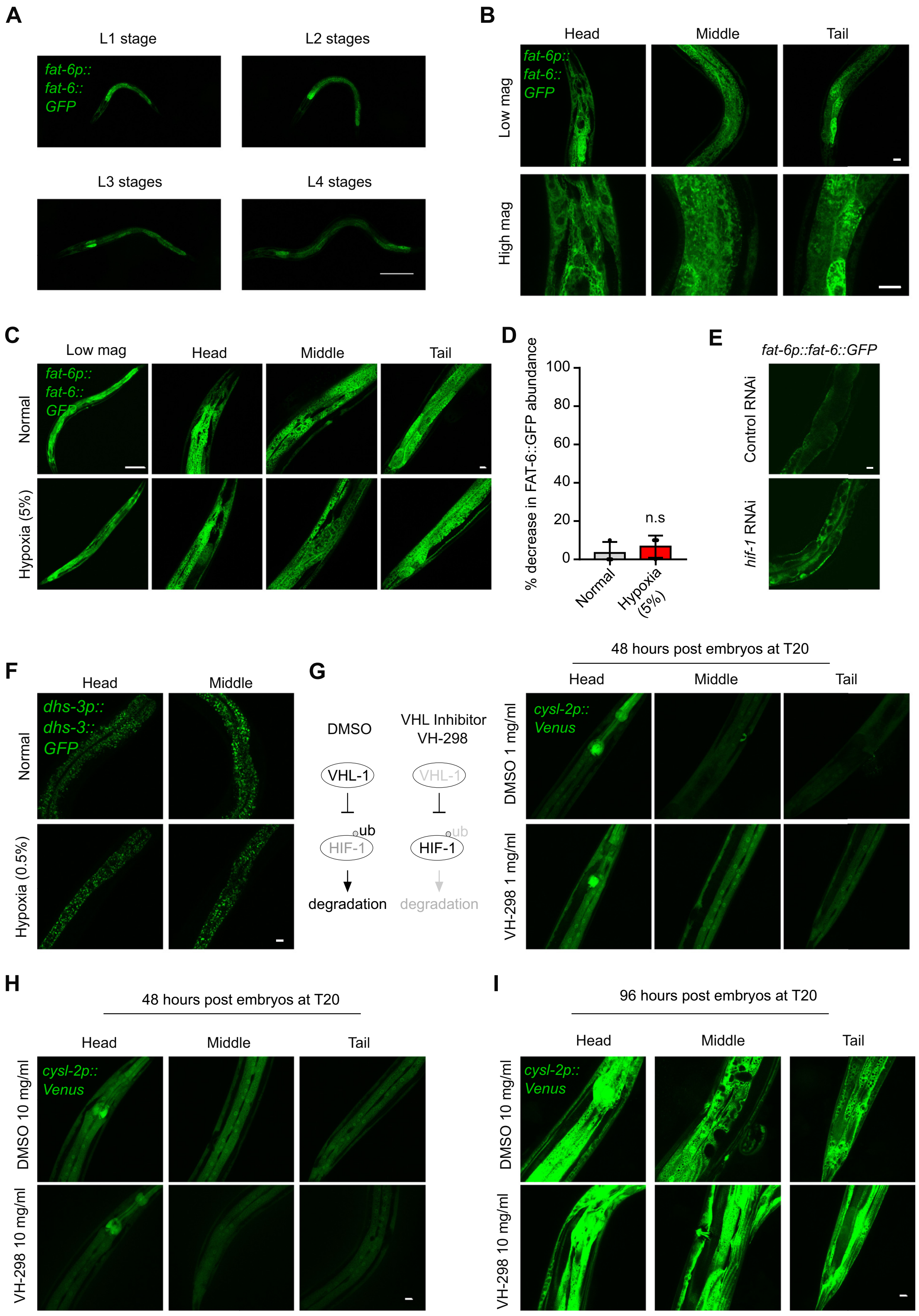
A
L1 stage
L2 stages
L3 stages
L4 stages
fat-6p::
fat-6::
GFP
B
Head
Middle
Tail
Low mag
High mag
fat-6p::
fat-6::
GFP
C
Low mag
Head
Middle
Tail
Normal
Hypoxia (5%)
fat-6p::
fat-6::
GFP
D
% decrease in FAT-6::GFP abundance
100
80
60
40
20
0
n.s
Normal
Hypoxia (5%)
E
fat-6p::fat-6::GFP
Control RNAi
hif-1 RNAi
F
Head
Middle
Normal
Hypoxia (0.5%)
dhs-3p::
dhs-3::
GFP
G
DMSO
VHL Inhibitor VH-298
VHL-1
ub
HIF-1
degradation
48 hours post embryos at T20
Head
Middle
Tail
DMSO 1 mg/ml
VH-298 1 mg/ml
cysl-2p::
Venus
H
48 hours post embryos at T20
Head
Middle
Tail
DMSO 10 mg/ml
VH-298 10 mg/ml
cysl-2p::
Venus
I
96 hours post embryos at T20
Head
Middle
Tail
DMSO 10 mg/ml
VH-298 10 mg/ml
cysl-2p::
Venus

Figure EV2. Characterization of expression patterns of FAT-6 and lipid droplets with various conditions in *C. elegans.*

(**A**) Representative confocal fluorescence images showing the expression of an integrated transgene *fat-6p::fat-6::GFP* under normal conditions at L1-L4 stages. Scale bars: 100 μm. (**B**) Representative Low and high magnification confocal fluorescence images showing the expression of *fat-6p::fat-6::GFP* under normal conditions L4 stages. Scale bars: 10 μm. (**C**) Representative confocal fluorescence images showing low and high-resolution Z-stack views of *fat-6p::fat-6::GFP* under normal or hypoxic (5%) conditions for 24 h post L4 stages. Scale bars: 10 μm. (**D**) Quantification of the percentage of animals with *fat-6p::fat-6::GFP* fluorescence intensities downregulated under normal or hypoxic (5%) conditions for 24 h post L4 stages. Data were presented as means ± S.D. *P* values calculated by unpaired two-tailed t-tests. n.s indicates nonsignificant ($n > 25$ animals per condition). (**E**) Representative confocal fluorescence images showing *fat-6p::fat-6::GFP* fed with control RNAi or RNAi against *hif-1*. Scale bars: 10 μm. (**F**) Representative confocal fluorescence images showing the downregulation of *dhs-3p::dhs-3::GFP*-labeled lipid droplet number and size under normal or hypoxic (0.5%) conditions for 24 h post-L4 stages. Scale bars: 10 μm. (**G**) Schematic diagram of HIF-1, VHL-1, and the drug VH-298. Representative confocal fluorescence images showing the upregulation of HIF-1 target reporter *cysl-2p::Venus* treat with DMSO or VHL-1 inhibitor VH-298 (1 mg/ml) for 48 h starting at embryos. Scale bars: 10 μm. (**H**) Representative confocal fluorescence images showing the upregulation of HIF-1 target reporter *cysl-2p::Venus* treat with DMSO or VHL-1 inhibitor VH-298 (10 mg/ml) for 48 h starting at embryos. Scale bars: 10 μm. (**I**) Representative confocal fluorescence images showing the upregulation of HIF-1 target reporter *cysl-2p::Venus* treat with DMSO or VHL-1 inhibitor VH-298 (10 mg/ml) for 96 h starting at embryos. Scale bars: 10 μm.

© The Author(s)

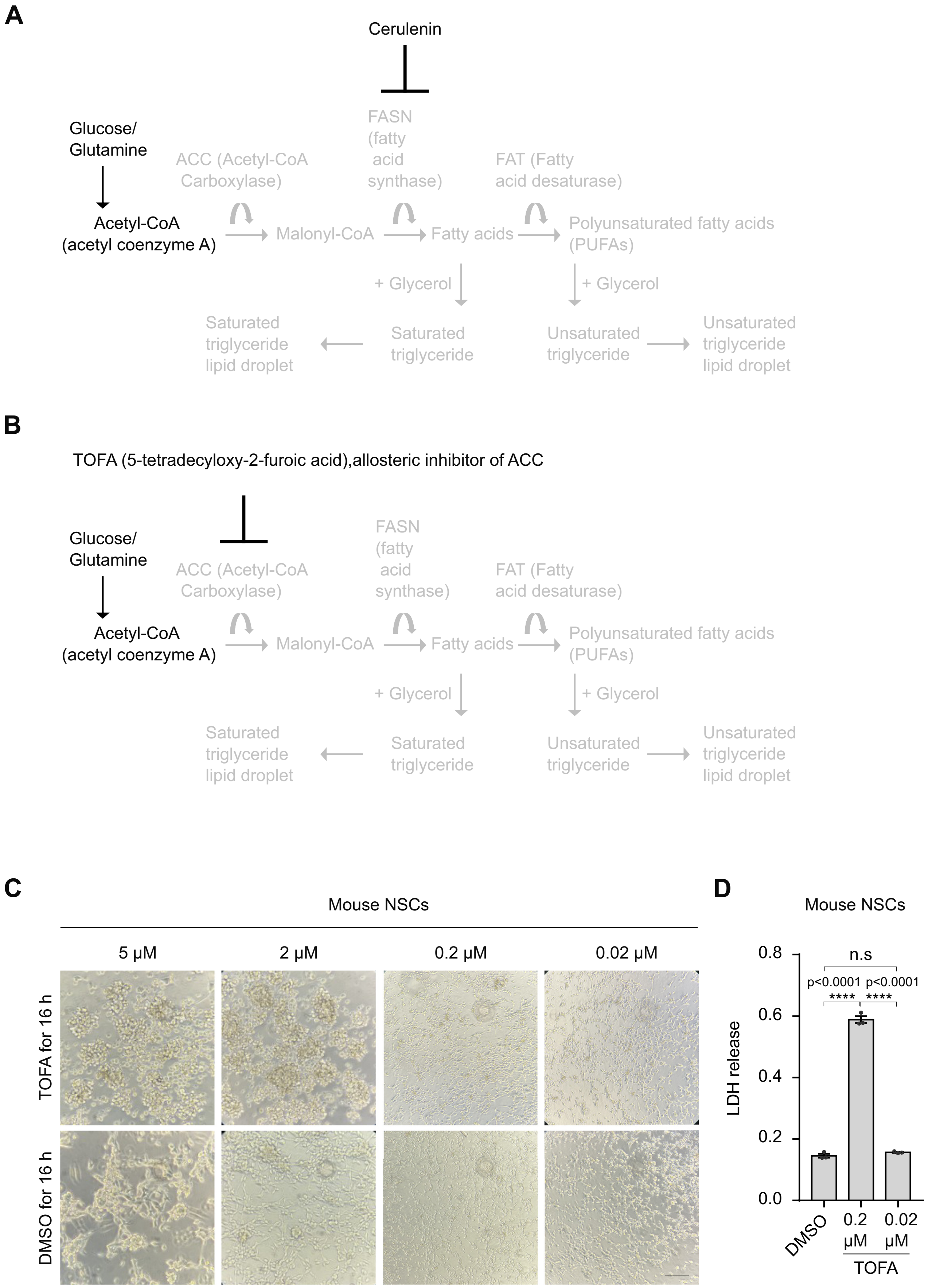
A
Cerulenin
Glucose/
Glutamine
Acetyl-CoA
(acetyl coenzyme A)
ACC (Acetyl-CoA
Carboxylase)
Malonyl-CoA
FASN
(fatty
acid
synthase)
Fatty acids
FAT (Fatty
acid desaturase)
Polyunsaturated fatty acids
(PUFAs)
+ Glycerol
+ Glycerol
Saturated
triglyceride
lipid droplet
Saturated
triglyceride
Unsaturated
triglyceride
Unsaturated
triglyceride
lipid droplet
B
TOFA (5-tetradecyloxy-2-furoic acid),allosteric inhibitor of ACC
Glucose/
Glutamine
Acetyl-CoA
(acetyl coenzyme A)
ACC (Acetyl-CoA
Carboxylase)
Malonyl-CoA
FASN
(fatty
acid
synthase)
Fatty acids
FAT (Fatty
acid desaturase)
Polyunsaturated fatty acids
(PUFAs)
+ Glycerol
+ Glycerol
Saturated
triglyceride
lipid droplet
Saturated
triglyceride
Unsaturated
triglyceride
Unsaturated
triglyceride
lipid droplet
C
Mouse NSCs
5 μM
2 μM
0.2 μM
0.02 μM
TOFA for 16 h
DMSO for 16 h
D
Mouse NSCs
n.s
p<0.0001 p<0.0001
**** ****
LDH release
0.0
0.2
0.4
0.6
0.8
DMSO
0.2 μM
0.02 μM
TOFA

◀ **Figure EV3. Characterization of TOFA in mouse neural stem cell.**

(**A**, **B**) Schematic of FASN inhibitor cerulenin and acetyl-CoA carboxylase (ACC) inhibitor TOFA (5-(Tetradecyloxy)-2-furoic acid), which blocks both saturated and unsaturated triglyceride biosynthesis. (**C**, **D**) Representative images and LDH release of mouse NSCs treated with different concentrations of TOFA or DMSO for 16 h. Data were presented as means ± SEM. P values calculated by one-way ANOVA. ****$P < 0.0001$, n.s indicates nonsignificant. $n = 3$ biological replicates. Scale bar: 100 μm.

 © The Author(s)

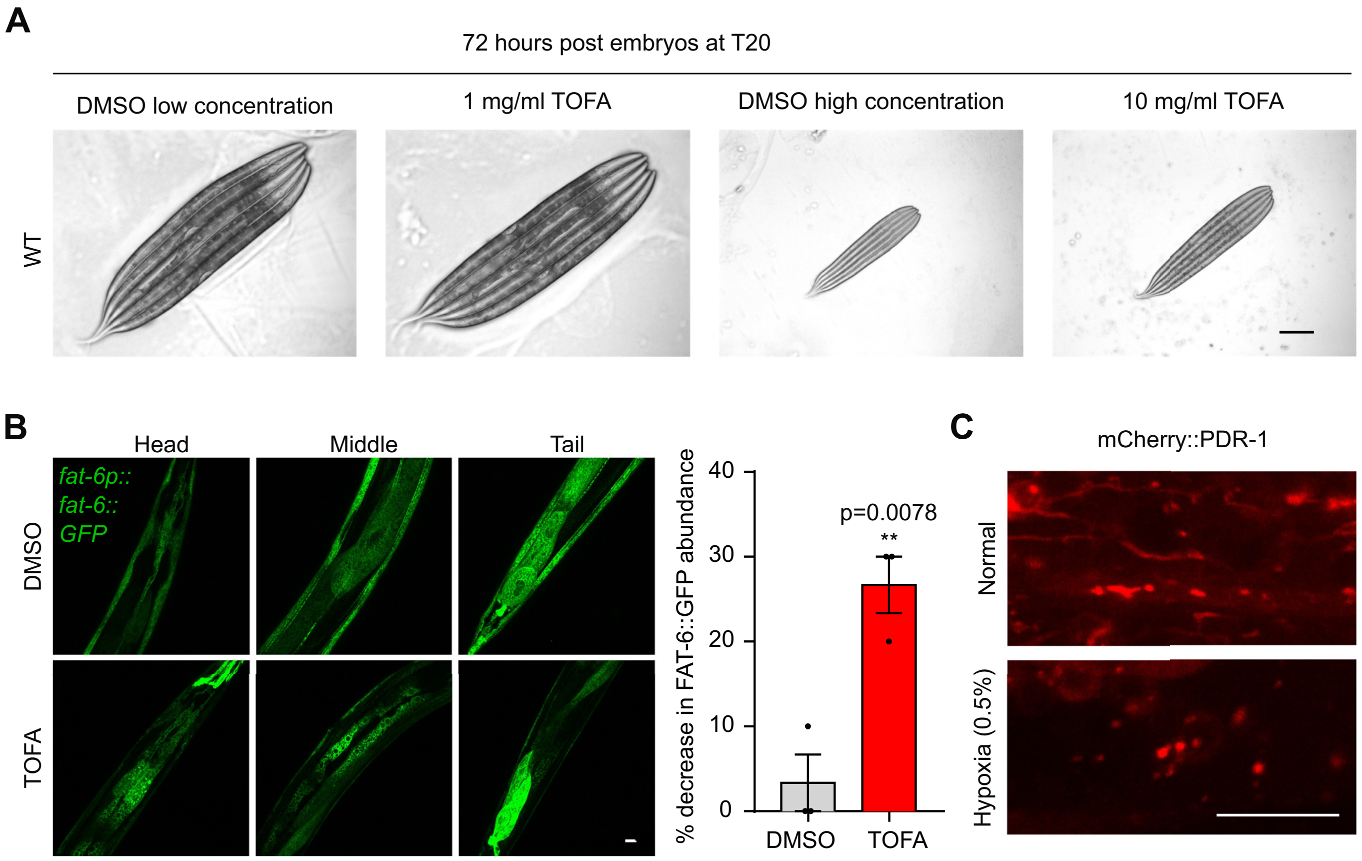

Figure EV4. Characterization of TOFA and expression patterns of mCherry::PDR-1 upon exposure to short-term hypoxia in *C. elegans*.

(**A**) Representative images of body size in wild-type N2 animals treated with varying concentrations of DMSO or TOFA, starting at the embryo stage at 20 °C. Scale bar: 100 µm. (**B**) Representative confocal fluorescence images and quantification of the percentage of animals with *fat-6p::fat-6::GFP* fluorescence intensities downregulated treated with DMSO or 1 mg/ml TOFA starting at the embryo stage. Scale bar: 10 µm. Data were presented as means ± S.D. *P* values calculated by unpaired two-tailed t-tests. $^{**}P < 0.01$ ($n = 30$ animals per condition). (**C**) Representative confocal fluorescence images showing high-resolution Z-stack tail area views of mCherry-PDR-1-labeled mitochondria morphology under normal or hypoxia (0.5%) for 24 h post-L4 stages. Scale bars: 10 µm.

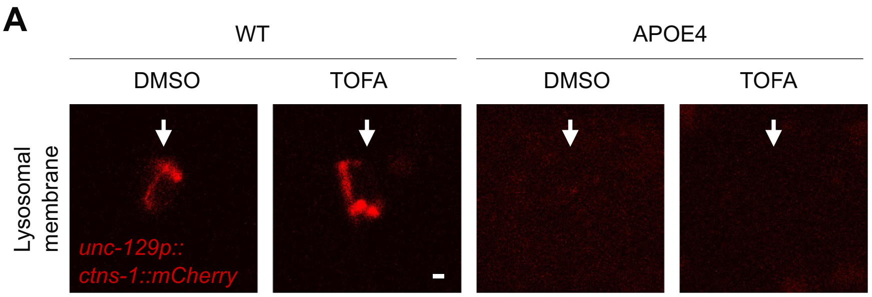

Figure EV5. TOFA cannot rescue APOE4-induced neuronal lysosomal abnormalities in *C. elegans.*

(**A**) Representative high magnification confocal microscopic images of neuronal specific lysosomal membrane reporter (white arrows) *ceIs56 [unc-129p::ctns-1::mCherry + nlp-21p::Venus + ttx-3p::RFP]*) at the YA stage, treated with DMSO or TOFA starting at the embryo stage. Scale bar: 1 µm.

 © The Author(s)

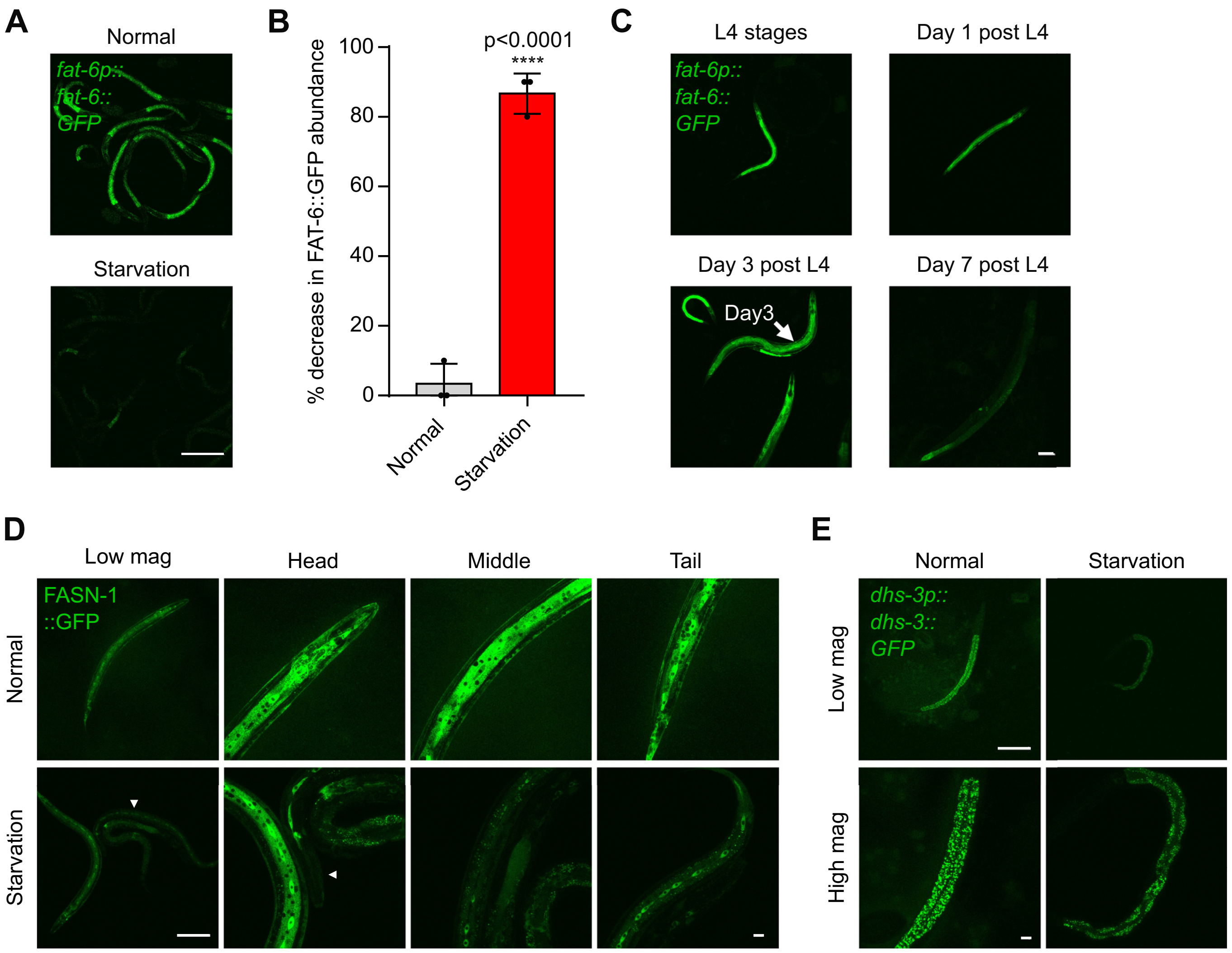

Figure EV6. FAT-6::GFP downregulation during starvation and aging in *C. elegans*.

(**A**) Representative confocal fluorescence images showing high-resolution Z-stack views of *fat-6p::fat-6::GFP* under 20 °C normal conditions or starvation of L1-L2 stages. Scale bars: 100 µm. (**B**) Quantification of the percentage of animals with *fat-6p::fat-6::GFP* fluorescence intensities downregulated under normal or starvation conditions. Data were presented as means ± S.D. *P* values calculated by unpaired two-tailed t-tests. *****P* < 0.0001 (*n* > 25 animals per condition). (**C**) Representative confocal fluorescence images showing low-resolution Z-stack views of *fat-6p::fat-6::GFP* under 20 °C normal conditions for 0 day, 1 day, 3 days or 7 days post L4 stages. Scale bars: 100 µm. (**D**) Representative confocal fluorescence images showing low and high-resolution Z-stack views of FASN-1::GFP under 20 °C normal conditions or starvation (white arrows indicate positively changed worms). Scale bars: 100 µm or 10 µm (magnification). (**E**) Representative confocal fluorescence images showing low and high-resolution Z-stack views of DHS-3::GFP under 20 °C normal conditions or starvation. Scale bars: 100 µm or 10 µm (magnification).

© The Author(s)
